# Where Are We Now with the Role of Steroids in the Management of Bronchopulmonary Dysplasia in Extremely Premature Babies?

**DOI:** 10.3389/fped.2016.00085

**Published:** 2016-08-10

**Authors:** Matthew Hurley, Jayesh Mahendra Bhatt

**Affiliations:** ^1^Nottingham Children’s Hospital, Nottingham, UK; ^2^University of Nottingham Division of Child Health, Nottingham, UK

**Keywords:** bronchopulmonary dysplasia, corticosteroids, prematurity, oxygen, neurodevelopment

The prevention and/or rescue of babies at risk of bronchopulmonary dysplasia (BPD) has focused upon the use of maternal antenatal steroids (1), surfactant (2), diuretics (3), and postnatal use of steroids along with various ventilator support strategies.

With regard to the evidence informing the use of steroids, there is no lack of data. Systemic corticosteroid administration (predominantly dexamethasone) through the immediate postnatal (4), moderate early (5) and late (6) periods, result in improved pulmonary outcomes (4–6) at a cost of short-term adverse effects (4). The use of corticosteroid treatment to treat or prevent development of established BPD remains controversial, however, largely due to concerns of neurodevelopmental sequelae. These are largely driven by one study (7), but as a whole, the inclusion of very varied steroid administration strategies in the same analyses make interpretation difficult.

Recently, a series of multicentre, randomized, adequately powered, controlled trials (summarized in Table 1) have examined the use of alternative corticosteroids [hydrocortisone or betamethasone (8)] at lower systemic doses (9) or lung administration either through nebulization (10) or direct instillation (11) to limit systemic exposure.

**Table 1:** **Trials of alternative route or dose of corticosteroids in BPD**.

Study	Patients	Intervention	Control	Outcome
Bassler et al. (10)	863 extremely preterm infants (<28 weeks gestation) requiring any positive pressure support in the first 12 h of life in centers in Europe and the US	441 infants were assigned to receive 400 μg budesonide 12 hourly for 14 days and 200 μg 12 hourly until no longer needing supplemental oxygen or reaching 32 weeks corrected gestation (whichever sooner)	422 infants were assigned to receive placebo 12 hourly until no longer needing supplemental oxygen or reaching 32 weeks corrected gestation (whichever sooner)	175 out of 437 (40%) infants who received budesonide died or had BPD compared to 194 out of 419 (46.3%) in the control group; OR 0.71, 95% CI 0.53–0.97, *p* = 0.03
Baud et al. (9)	523 extremely preterm infants inborn (born in a maternity ward at the same site as the NICU) at less than 28 weeks of gestation at 21 French centers	255 infants received 1 mg/kg hydrocortisone per day divided into two doses per day for 7 days, followed by one dose of 0·5 mg/kg per day for 3 days	267 infants received placebo (5% dextrose)	A greater proportion of infants who received hydrocortisone, 153 out of 255 (60%) survived without BPD, compared with 136 (51%) out of 266 infants assigned to placebo. OR (adjusted for gestational age group and interim analyses) 1·48, 95% CI 1·02–2·16, *p* = 0·04. NNT 12, 95% CI 6–200
Nakamura et al. (12)	211 infants with birthweight <1000 g who needed intubation for respiratory support in Japan	107 infants received two doses of 50 μg of Fluticasone or placebo were administered every 24 h *via* the spacer device starting within 24 h of birth and continuing until 6 weeks of age or extubation	104 infants received placebo	No difference between groups in incidences of death or oxygen dependence at discharge (RR 0.94, 95% CI 0.72–1.23). The 24–26 GA subgroup analyses demonstrated that death and oxygen dependence at discharge were significantly lower in the Fluticasone group (RR 0.43, 95% CI 0.20–0.92)
Yeh et al. (11)	265 very-low-birth-weight infants (<1500 g) in the US & Taiwan with severe RDS who required mechanical ventilation and FiO_2_ >50% within 4 h of birth	131 infants received surfactant (100 mg/kg) and budesonide (0.25 mg/kg) 8 hourly until FiO_2_ <30%	131 infants received surfactant (100 mg/kg). Only 8 hourly until FiO_2_ <30%	Infants in the budesonide group had a lower incidence of BPD or death [55 out of 131 (42.0%) vs. 89 out of 134 (66%) respectively; RR, 0.58, 95% CI 0.44–0.77, *p* = 0.001; NNT 4.1 95% CI 2.8–7.8]

The PREMILOC study (9) of low-dose hydrocortisone (1 mg/kg for 7 days with a 3-day wean; 8.5 mg/kg cumulative dose) recruited 523 extremely premature infants within the first day of life. The primary outcome was survival without BPD at 36 weeks of post menstrual age. The trial was stopped early due to “financial and technical support limitations,” and the planned analysis was adjusted as a result. 153 (60%) out of 255 infants assigned to receive hydrocortisone and 136 (51%) out of 266 infants assigned to placebo, survived without BPD. The effect size, adjusted for gestation, and the effect of repeated analyses in the statistical method was statistically significant – OR 1·48 (95% CI 1·02–2·16; *p* = 0·04). The number needed to treat (NNT) to achieve one BPD-free survival was 12 (95% CI 6–200). There was a significantly increased risk of late onset sepsis in those born at 24–25 weeks gestation given hydrocortisone (33/83; 40%) compared with placebo (21/90; 23%). Unfortunately, long-term neurodevelopmental outcome is not yet reported; however, MRI findings as a surrogate were available in 80% of the survivors with 30% of infants in each group demonstrating detectable lesions at term-corrected gestations.

The NEUROSIS trial (10) of inhaled budesonide (400 μg twice daily *via* a spacer for 2 weeks, then once daily until supplemental oxygen was not required or infant reached 32 weeks PMA) or placebo recruited 863 infants who needed respiratory pressure support in 40 centers in 9 European countries. Primary outcome was identical to the PREMILOC study (except rate of death or BPD at 36 weeks PMA). In a per-protocol analysis of 437 infants randomized to receive budesonide, 175 (40%) died or developed BPD compared with 194 out of 419 (46.3%) infants in the placebo group (effect size OR 0.71; 95% CI 0.53–0.97; *p* = 0.03). Those receiving budesonide appeared to come out of oxygen earlier and fewer babies required re-intubation. Unfortunately, neurodevelopmental outcomes are as yet unreported. In contrast, these were reported in a multicentre trial from Japan, where infants were randomized to receive inhaled fluticasone (*n* = 107) or placebo (*n* = 104). No significant differences were detected between the groups with respect to either the frequency of death, oxygen dependence at discharge, or neurodevelopmental impairment at 18 months and 3 years PMA. In subgroup analyses, the frequencies of death and oxygen dependence at discharge were significantly decreased in the fluticasone group for infants born at 24–26 weeks and for infants with chorioamnionitis, regardless of the gestational age at birth (12).

In the study by Yeh et al. (11), 265 very-low-birth-weight (<1500 g) infants with severe respiratory distress syndrome requiring invasive ventilation were randomised to receive either intratracheal surfactant alone or in a combination mixed with budesonide (0.25 mg or 1 ml/kg). Doses were repeated every 8 h until infants, required less than 30% oxygen, were extubated or had received a maximum of six doses. The primary outcome measure was again identical to the two other studies. Infants in the combined surfactant-budesonide group had a lower incidence of BPD or death (55 out of 131, 42%) compared with those in the surfactant-only group (89 out of 134, 66%) (RR 0.58; 95% CI 0.44–0.77; *p* < 0.001). The NNT was 4.1 (95% CI 2.8–7.8). With 85% of the cohort under follow-up at 30 months of age, the proportions of infants described as having “neurodevelopmental impairment” were 26 out of 85 (30.6%) in the intervention group compared with 34 out of 87 (39.1%) in the control group, with follow-up ongoing.

The four recent studies report the use of local/lower dose corticosteroid to prevent BPD/death and find a significant beneficial effect, at least in selected groups of babies. This is unsurprising as efficacy has really not been in doubt, although, it is encouraging that a reduced dose exerts a similar effect. It is disappointing that, as yet, the factor limiting adoption, neurodevelopmental impairment, has not been reported in three of these studies, and these studies are unlikely to change practice until it is.

While these data add to evidence for preventing BPD, there remains a large void in the evidence base informing the treatment of those with established severe BPD. Indeed, the current studies appear to show no promise for this small group of infants as the proportion of infants with very severe BPD was no different between treatment and control groups in the PREMILOC study (9). Management for these infants is likely to vary to an even greater degree than the approach taken across units or countries (13) with the use of steroids for all premature infants (14). One approach extrapolates the strategy used for childhood interstitial lung disease (15) but restricts it to those considered to have life-threateningly very severe BPD with an objective measure of severity.

While the studies that have been completed need follow-up in order to report neurodevelopmental outcome, the pendulum does appear to have come back toward the center in terms of steroid use in preventing BPD. In the case of established, very severe BPD, it is not just a case of “more studies are needed,” one study would be a start.

## Author Contributions

JB conceived the idea and provided the structure of the commentary. MH wrote the first draft. Both authors agreed the final draft.

## Conflict of Interest Statement

The authors declare that the research was conducted in the absence of any commercial or financial relationships that could be construed as a potential conflict of interest.
